# A case of neuropsychiatric lupus Erythematosus characterized by the Owl’s eye sign: a case report

**DOI:** 10.1186/s12883-017-0902-6

**Published:** 2017-06-29

**Authors:** Bolin Hu, Pengcheng Wu, Yibiao Zhou, Yan Peng, Xiaoping Tang, Weijiang Ding, Ming Zhang, Xueliang Qi

**Affiliations:** 1 Department of Neurology, The Second Affiliated Hospital of Nanchang University, No. 1, Minde Road, Nanchang, Jiangxi 330006 People’s Republic of China; 2 Department of Orthopedics, The First Affiliated Hospital of Nanchang University, Nanchang, Jiangxi 330006 People’s Republic of China; 3 Department of MRI, The Second Affiliated Hospital of Nanchang University, Nanchang, Jiangxi 330006 People’s Republic of China

**Keywords:** Neuropsychiatric lupus, Magnetic resonance imaging, Owl’ eye sign, Case report

## Abstract

**Background:**

Systemic lupus erythematosus (SLE) is an autoimmune inflammatory disorder characterized by multiple affected systems. More than half of SLE patients will suffer from neuropsychiatric lupus erythematosus (NPSLE) during the course of their disease. Although nearly half of the NPSLE patients have normal MRI manifestations, the abnormalities found in the remainder can be located anywhere in the brain, and especially in the subcortical white matter of the frontal and temporal lobe. However, NPSLE involving the medulla oblongata and spinal cord which presents as the “owl’s eye” sign has to our best knowledge not been reported to date.

**Case presentation:**

A 19-year-old girl presented at our hospital with a 7-day history of fever and headache since a one day’s exertion, accompanied by 2 days of weakness. The patient had slurred speech. Neurological examination revealed the presence of horizontal nystagmus and a limitation of bilateral eye movement when looking up and down. At the same time, she showed difficulty in raising the jaw, accompanied by a weak pharyngeal reflex. Muscle strength was remarkably decreased in all four extremities: the MRCS grade of the upper limbs was 4/5, while in the lower limbs it was 0/5. Hypotonia was apparent in the lower extremities. Regarding subjective sensation, the patient appeared to be experiencing an increased sense of pain in the whole body, and especially in the cervical region, abdomen, and feet. An examination of shallow reflex documented the reinforcement of the abdominal reflex. Deep tendon reflexes were symmetric: absent in lower, normal in upper extremities. The patient also had a stiff neck with a positive Kernig’s sign. The laboratory examination showed elevated C - reactive protein and rheumatoid factor, as well as complement components 3 and 4. Symptomatic treatments were applied, but she did not respond well, after which we did immunological laboratory examinations. The results showed the presence of anti-nRNP/Sm, anti-dsDNA and anti-AMA M2 antibodies. An MRI scan and enhancement of the cervical and thoracic regions displayed abnormal signs in the medulla and bilateral anterior horn of the lower thoracic spine. Following the exclusion of other possible diseases, neuropsychiatric lupus was diagnosed. High-dose intravenous gamma-globulin combined with methylprednisolone gradually improved her condition.

**Conclusion:**

We report the first case of NPSLE presenting with medulla oblongata and spinal cord involvement, manifesting as the “owl’s eye” sign in MRI.

## Background

Systemic lupus erythematosus (SLE) is an autoimmune inflammatory disorder characterized by multiple affected systems [1]. More than half of SLE patients will suffer from neuropsychiatric lupus erythematosus (NPSLE) during the course of their disease [2, 3]. An exact definition of NPSLE is challenging because of the broad spectrum of its manifestations. In 1999 NPSLE was classified by the American College of Rheumatology (ACR), which identified 19 neuropsychiatric syndromes including 12 central nervous system (CNS) forms and 7 peripheral nervous system forms [4]. Even though nearly half of all NPSLE patients have a normal MRI presentation [5], the abnormalities in the other patients can be found anywhere in the brain, and especially in the subcortical white matter of the frontal and temporal lobe [6]. However, NPSLE with medulla oblongata and spinal cord involvement which presents as the “owl’s eye” sign has to our best knowledge not been reported before. We therefore report the first case of NPSLE with medulla oblongata and spinal cord involvement, manifesting as the “owl’s eye” sign in MRI.

## Case presentation

A 19-year-old girl was admitted to our hospital with a 7-day history of fever and headache since an episode of one day’s exertion, and two days of weakness. Seven days before clinical presentation, the girl had developed a headache, sore throat, bucking, vomiting and fever after one day’s hard work. Consequently, she went to the local hospital, and after several days of symptomatic treatment, only the temperature dropped slightly. She therefore came to our hospital for further diagnosis and treatment. The patient had a history of recurring joint swelling, chilblains and alopecia. Her personal history was unremarkable, and no similar symptoms were found in her family. When she was transferred to our hospital, she was in a state of sleepiness, dysarthria, dysphagia and fever, with a body temperature of 37.8 °C, while an examination of the heart, lungs and abdomen revealed that they were normal. A neurological examination revealed a horizontal nystagmus and a limitation of bilateral eye movement when looking up and down. She also showed difficulty in raising the jaw, accompanied by a weak pharyngeal reflex. Muscle strength was remarkably decreased in all four extremities, with upper limbs having an MRCS grade of 4/5, while it was 0/5 in the lower limbs. Hypotonia was apparent in the lower extremities. Regarding the patient’s subjective sensation, she appeared to exhibit an increased sense of pain in the whole body, and especially in the cervical region, abdomen, and feet. An examination of shallow reflex documented a reinforcement of the abdominal reflex. Deep tendon reflexes were symmetric: absent in the lower, normal in the upper extremities. The patient also had a stiff neck with a positive Kernig’s sign.

**Fig. 1:**
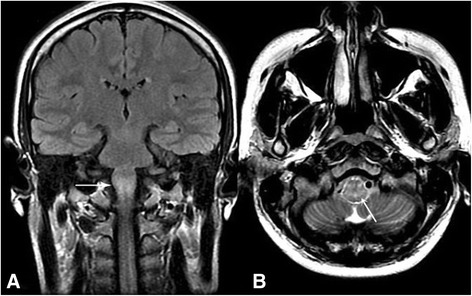
**a** and **b** coronal T2 flair magnetic resonance and axial T2 weighted imaging showing diffuse swelling and high signal intensity involving the medulla oblongata

**Fig. 2:**
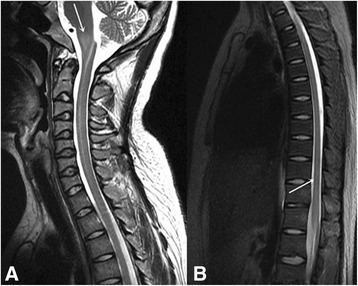
**a** and **b** sagittal T2 weighted imaging showing high signal intensity involving the medulla oblongata and thoracic spinal cord

**Fig. 3:**
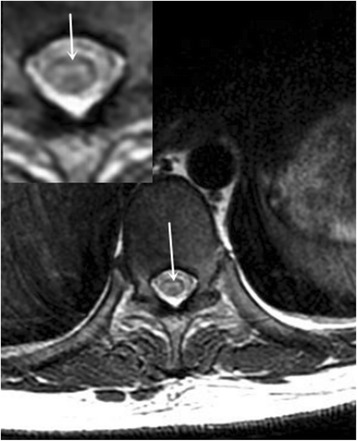
Axial T2 weighted imaging and magnified image of thoracic spinal cord, showing a small rough high signal appearing in the bilateral anterior horn presenting as the “owl’s eye” sign

Laboratory examination revealed elevated inflammatory indicators, with a white blood cell count of 8.41 × 10^9^/L(normal range 4–10 × 10^9^/L), NE% of 87.8%(normal range 50–70%), erythrocyte sedimentation rate of 72 mm/h(normal range 0-20 mm/h), and C-reactive protein concentration of 44.50 mg/L(normal range 0–8 mg/L). Quantitative serum analysis revealed the presence of 676.21 IU/ml of anti-dsDNA antibodies (normally < 100 IU/ml), with a titer of 1:40. Furthermore, the results for the anti-nRNP/Sm, anti-dsDNA, anti-ANuA, anti-AMA M2 antibody and IgM antiphospholipid antibody were all positive (normal being negative). The titer of the speckled anti-nuclear antibody was 1:640. The results of quantitative analysis for serum complement components 3 and 4 were 0.47 g/L and 40.05 g/L, respectively (normal range 0.9–1.8 g/L and 0.1–0.4 g/L, respectively). Simultaneously, rheumatoid factors were present at 23.70 IU/m (normally < 20 IU/m). Lumbar puncture revealed a cerebrospinal fluid pressure of 220mmH_2_O (normal range 80–180 mmH_2_O), with elevated protein at 775.79 mg/L (normal range 150-450 mg/L), IgG at 224.00 mg/L (normal range 0–34 mg/L), and a leukocyte number of 110 × 10^6^/L (normal range 1.5–4.0 × 10^9^/L). By contrast, tests for tumor cells and bacterial, viral, or fungal infection of CSF were all negative. Other indicators, including anti-aquaporin 4 antibody, the serum IgG oligoclonal band, and antibodies related to paraneoplastic symptoms of CSF were negative (normal). The EEG documented a significant amount of bilateral hemispheric slow waves with a moderate amplitude. The MRI comprising coronal T2 flair magnetic resonance and axial T2 weighted imaging showed diffuse swelling and high signal intensities involving the medulla oblongata **(**Fig. 1a and b. On sagittal T2 weighted imaging, high intensity signals were found in the medulla oblongata and thoracic spinal cord (Fig. 2a and b). In addition, axial T2 weighted imaging and the magnified image of the thoracic spinal cord revealed a small rough high signal in the bilateral anterior horn showing an“owl’s eye” sign **(**Fig. 3. At the same time, CT angiography from the cervical region to the skull base was normal. According to the clinical features, laboratory examination and imaging results, SLE was confirmed, and after ruling out other possible diseases, NPSLE was finally diagnosed. Intravenous immunoglobulin (400 mg/kg, Qd, for 5 days), methylprednisolone (1000 mg/day for 3 days, followed by 500 mg/day for another 3 days, and 250 mg/day for another 3 days), cyclophosphamide (400 mg weekly), and aspirin (100 mg/day) were administered for therapy. The patient’s eye movement, dysarthria and dysphagia recovered obviously within a week. The mobility of the soft palate normalized completely and the muscle strength of the lower limbs improved to grade 2/5 within 22 days. At the 3-month follow-up, the patient’s neurological deficit was completely resolved with weekly i.v. pulsed cyclophosphamide (400 mg/week) and prednisolone (20 mg/day). From then on, the patient was able to walk independently.

## Discussion

NPSLE encompasses a number of neurological or psychiatric symptoms in which the SLE involves the central or peripheral nervous system. When NPSLE is considered, SLE should be diagnosed primarily, and possible non-NPSLE diseases such as the side-effects of medicines, mental or functional conditions should be excluded. In various studies, about 28% to 40% of adult SLE patients were found to develop NPSLE before or during the diagnosis of SLE, and about 63% developed NPSLE within one year after the diagnosis of SLE [6]. Several imaging modalities can be used to investigate NPSLE. Owing to its sensitivity, availability, and ability to exclude various similar conditions, MRI is the method of choice for the evaluation of NPSLE [5, 7]. Both longitudinal and transverse myelitis have been reported in SLE, and they represent well-characterized neurological manifestations of the disorder [8, 9]. However, NPSLE with medulla oblongata and spinal cord involvement which presents as the “owl’s eye” sign has to our best knowledge not been reported to date.

The main pathophysiological changes found in NPSLE are vascular lesions characterized by hyaline degeneration, proliferation of endothelial cells and gliosis around vessels [10]. However, the underlying pathophysiological mechanisms of NPSLE remain unknown. Nevertheless, it is known that a disruption of the function of the blood-brain barrier may play a role in the development of NPSLE [11], and autoantibodies complement components, and cytokines are integral to the pathogenesis pathways, with more than 20 autoantibodies reported to be linked to NPSLE [12]. Furthermore, several studies have shown that cerebrovascular diseases and transverse myelitis in SLE are strongly related to the antiphospholipid antibodies, which accelerate atherosclerosis and thrombosis [13–15]. Regrettably, the presence of autoantibodies was not analyzed in this patient.

Medulla oblongata and spinal cord involvement has been reported in several cases. Neuman-Andersen et al. found swelling and signal changes in the medulla oblongata and the entire spinal cord, especially in the lower thoracic cord and conus [16]. Kimura et al. discovered a longitudinal lesion spanning the region from the medulla oblongata, C6 and Th12, reaching to the conus [17]. Mimenza-Alvarado et al. also found brain stem disorders in SLE patients [18]. Although medulla and/or spinal cord involvement was found in said cases, the changes included neither diffuse swelling nor increased signal density in the sagittal and/or axial MRI. Luckily, we have the honor of having discovered a unique change in neuropsychiatric lupus erythematosus, which presented as the “owl’s eye” sign in the MRI.

The “owl’s eye” sign, or alternatively “snake-eye” sign, is a specific finding in neuroimaging characterized by bilaterally symmetrical high signal intensity in the anterior horn of the spinal cord in axial T2-weighted MR imaging, which evokes the eyes of an owl or snake [19]. It has been reported in a number of diseases, such as complex cervical spondylosis, Hirayama disease, poliomyelitis, encephalitis caused by HIV infection, spinal cord infarction, neuromyelitis optica, paraneoplastic neurological syndrome and flail arm syndrome [20, 21]. Neuroimaging-documented appearance of the “owl’s eye” sign may reflect cystic necrosis in the central gray matter near the ventrolateral posterior column, and is consequently regarded as an unfavorable prognostic factor for the recovery of upper-extremity motor weakness, due to the destruction of gray matter accompanied by significant neuronal loss in the anterior horn [22].

After the initial 7-day history of fever and headache, our patient quickly developed catastrophic flaccid paralysis of the legs, accompanied by areflexia, disordered eye movement, dysarthria, and dysphagia within 24 h. This case is characterized by involvement of both medulla oblongata and spinal cord, which presented as the “owl’s eye” sign. In contrast to spinal cord infarction and similar diseases, which also lead to an “owl’s eye” sign, the prognosis of NPSLE is good with timely treatment. It is therefore necessary to consider a diagnosis of NPSLE early if the patient does not respond well to the non-NPSLE treatment.

## Conclusions

In summary, the case reported here presented with typical medulla oblongata and spinal cord involvement, which is rare in NPSLE patients. It therefore illustrates that NPSLE may involve the medulla oblongata and spinal cord, manifesting as the “owl’s eye” sign in MRI. The owl’s eye sign can thus be present in yet another disease, in addition to the many known examples such as complex cervical spondylosis, Hirayama disease, poliomyelitis, encephalitis caused by HIV infection, spinal cord infarction, neuromyelitis optica, paraneoplastic neurological syndrome and flail arm syndrome.

## Abbreviations

CRP: C-reactive protein
CSF: Cerebrospinal fluid
CT: Computed tomography
EEG: Electroencephalogram
IgG: Immunoglobulin G
IgM: Immunoglobulin M
MRI: Magnetic resonance imaging
NPSLE: Neuropsychiatric systemic lupus erythematosus
SLE: Systemic lupus erythematosus
